# Supplementary material for: Meta-Analysis: Urinary Calprotectin for Discrimination of Intrinsic and Prerenal Acute Kidney Injury
Source: J Clin Med. 2019 Jan 10;8(1):74. doi: 10.3390/jcm8010074 (PMC6352097; doi:10.3390/jcm8010074)
Supplement: Supplementary file 1 [file jcm-08-00074-s001.pdf]

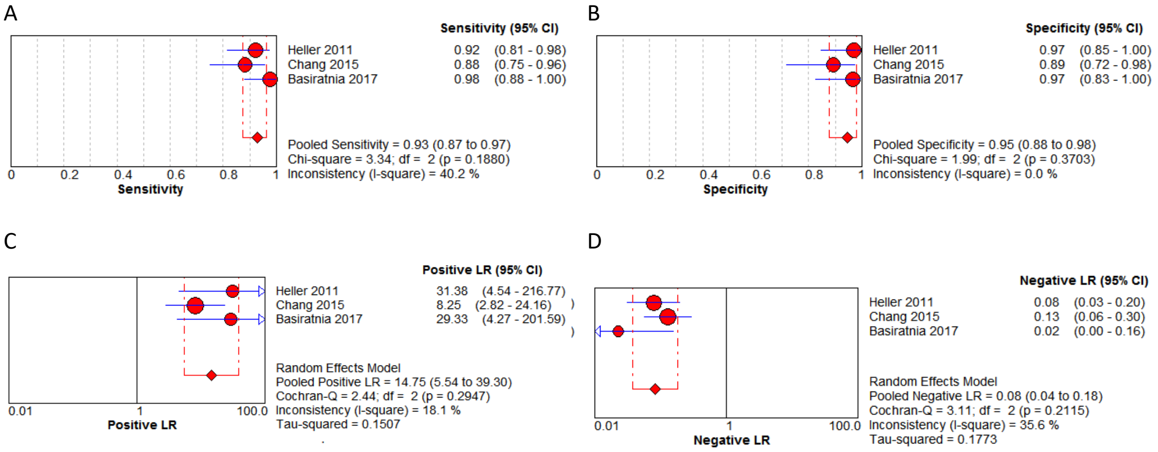

Figure S1. Diagnostic performance of the three studies providing data on urinary calprotectin with normalization to urine creatinine.

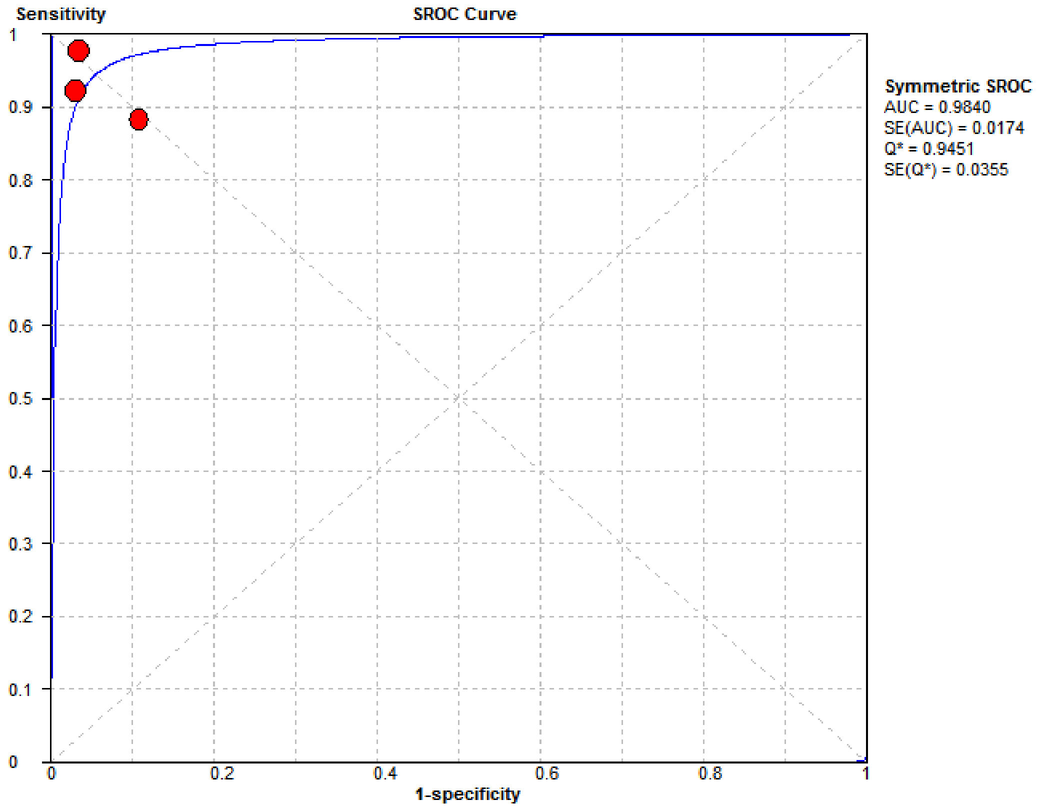

Figure S2. Symmetric SROC according to the cutoffs of the three studies with urinary calprotectin with normalization to urine creatinine. Abbreviation: SROC, summary receiving operating characteristics; AUC, area under the curve; SE, standard error.

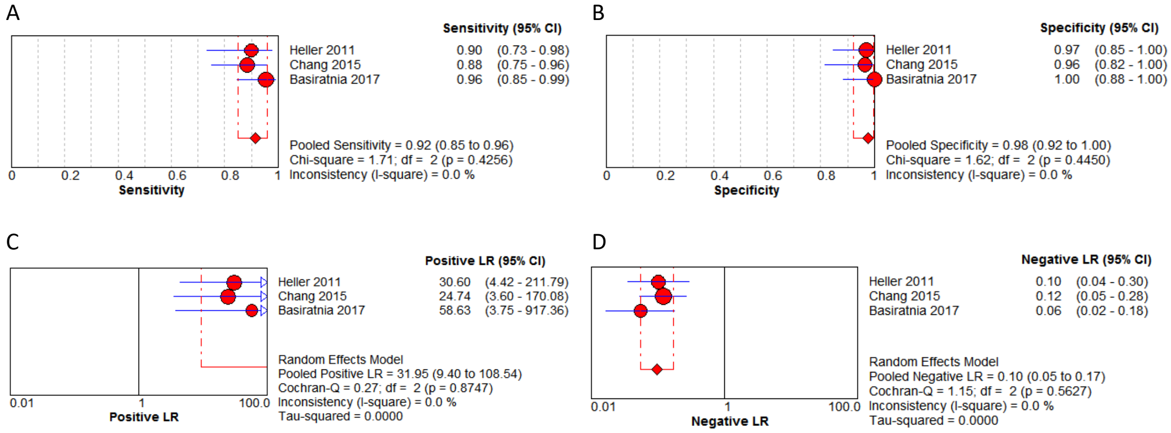

Figure S3. Diagnostic performance of urinary calprotectin with three studies excluding patients with urinary tract infection.

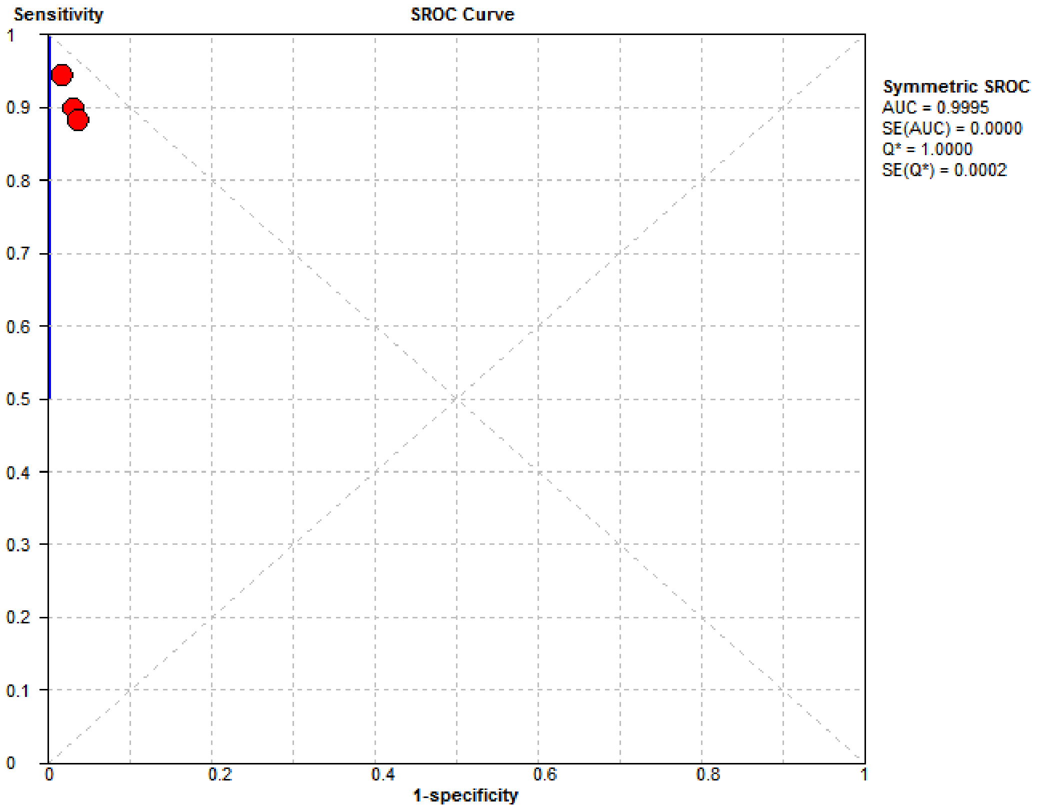

Figure S4. Symmetric SROC according to the cutoffs of the three studies excluding patients with urinary tract infection. Abbreviation: SROC, summary receiving operating characteristics; AUC, area under the curve; SE, standard error.

Table S1. Primary reasons for exclusion of excluded studies.

First Author	Last Author	Journal	Year	Title	Primary Reason for Exclusion	Include
Azimi, A.		Medical Hypotheses	2017	Could ''calprotectin'' and ''endocan'' serve as ''Troponin of Nephrologists''?	Review article	N
Alexander, F.	Jens, H.W.	Pediatric Nephrology	2017	Urinary biomarkers of acute kidney injury in very low birth weight infants on indomethacin for patent ductus arteriosus	Not distinguish intrinsic and pre-renal acute kidney injury	N
Chang, C.H.	Chen, Y.C.	Medicine	2015	Urinary Biomarkers Improve the Diagnosis of Intrinsic Acute Kidney Injury in Coronary Care Units	Include	Y
Dessing, M.C.	Leemans, J.C.	Kidney International	2015	The calcium-binding protein complex S100A8/A9 has a crucial role in controlling macrophage-mediated renal repair following ischemia/reperfusion	Not human study	N
Dobrek, L.	Thor, P.	Polish Annals of Medicine	2017	Novel biomarkers of acute kidney injury and chronic kidney disease	Review article	N
Ebbing, J.	Westhoff, T.H.	World Journal of Urology	2014	Urinary calprotectin: a new diagnostic marker in urothelial carcinoma of the bladder	Not acute kidney injury study	N
Ebbing, J.	Westhoff, T.H.	PLoS One	2016	Dynamics of Urinary Calprotectin after Renal Ischaemia	Not acute kidney injury study	N
Fan, P.C.	Chen, Y.C.	Nephrology (Carlton)	2018	Biomarkers for acute cardiorenal syndrome	Review article	N
Fujigaki, Y.	Yasuda, H.	Japanese Journal of Nephrology	2012	Acute kidney injury	Review article	N
Gao, S.	Liu, G.	American Journal of Emergency Medicine	2015	Diagnostic and prognostic value of myeloid-related protein complex 8/14 for sepsis	Not urinary calprotectin	N
Heller, F.	Westhoff, T.H.	Clinical Journal of the American Society of Nephrology	2011	Urinary calprotectin and the distinction between prerenal and intrinsic acute kidney injury	Include	Y

Ikemoto, M.	Fujita, M.	Clinica Chimica Acta	2007	Intrinsic function of S100A8/A9 complex as an anti-inflammatory protein in liver injury induced by lipopolysaccharide in rats	Not human study	N
Kim, A.J.	Jung, J.Y.	PLoS One	2016	Klotho and S100A8/A9 as Discriminative Markers between Pre-Renal and Intrinsic Acute Kidney Injury	Not human study	N
Kashani, K.	Ronco, C.	Clinical Chemistry and Laboratory Medicine	2017	Biomarkers of acute kidney injury: the pathway from discovery to clinical adoption	Review article	N
Lee, C.W.	Tsai, H.I.	World Journal of Emergency Surgery	2018	A combination of SOFA score and biomarkers gives a better prediction of septic AKI and in-hospital mortality in critically ill surgical patients: a pilot study	Not distinguish intrinsic and pre-renal acute kidney injury	N
Basiratnia, M.	Hooman, N.	Iranian Journal of Pediatrics	2017	Urinary calprotectin as a marker to distinguish functional and structural acute kidney injury in pediatric population	Include	Y
Ostermann, M.	Forni, L.G.	Critical Care	2012	Clinical review: Biomarkers of acute kidney injury: where are we now?	Review article	N
Ortega-Loubon, C.	Fulquet-Carreras, E.	Annals of Cardiac Anaesthesia	2016	Cardiac surgery-associated acute kidney injury	Review article	N
Plebani, M.		Clinical Chemistry and Laboratory Medicine	2017	Biomarkers of acute kidney injury: a step forward	Review article	N
Rekers, N.V.	Eikmans, M.	American Journal of Transplantation	2016	Beneficial Immune Effects of Myeloid-Related Proteins in Kidney Transplant Rejection	Not acute kidney injury study	N
Szeto, C.C.	Li, P.K.	Clinical Journal of the American Society of Nephrology	2010	Urinary expression of kidney injury markers in renal transplant recipients	Not acute kidney injury study	N
Schrezenmeier, E.V.	Schmidt-Ott, K.M.	Acta Physiologica (oxford)	2017	Biomarkers in acute kidney injury—pathophysiological basis and clinical performance	Review article	N
Seibert, F.S.	Westhoff, T.H.	Acta Physiologica (oxford)	2013	Calprotectin and neutrophil gelatinase–associated lipocalin in the differentiation of pre-renal and intrinsic acute kidney injury	Include	Y
Seibert, F.S.	Westhoff, T.H.	Transplantation	2016	Urinary Calprotectin Differentiates Between Prerenal and Intrinsic Acute Renal Allograft Failure	Include	Y

Seibert, F.S.	Westhoff, T.H.	Kidney & blood pressure research	2018	Prognostic Value of Urinary Calprotectin, NGAL and KIM-1 in Chronic Kidney Disease	Not acute kidney injury study	N
Tan, X.	Lin, Y.	Cellular Physiology and Biochemistry	2017	Involvement of S100A8/A9-TLR4-NLRP3 Inflammasome Pathway in Contrast-Induced Acute Kidney Injury	Not human study	N
Tepel, M.	Westhoff, T.H.	PLoS One	2014	Urinary calprotectin and posttransplant renal allograft injury	Not acute kidney injury study	N
Vanmassenhove, J.	Ostermann, M.	Intensive Care Medicine	2017	Have renal biomarkers failed in acute kidney injury? Yes	Review article	N
Westhoff, J.H.	Westhoff, T.H.	Pediatric Nephrology	2016	Urinary biomarkers for the differentiation of prerenal and intrinsic pediatric acute kidney injury	Include	Y
Westhoff, J.H.	Westhoff, T.H.	European Journal of Pediatrics	2017	Urinary calprotectin, kidney injury molecule-1, and neutrophil gelatinase-associated lipocalin for the prediction of adverse outcome in pediatric acute kidney injury	Duplication cohort	N
